# Coracle—a machine learning framework to identify bacteria associated with continuous variables

**DOI:** 10.1093/bioinformatics/btad749

**Published:** 2023-12-20

**Authors:** Sebastian Staab, Anny Cardénas, Raquel S Peixoto, Falk Schreiber, Christian R Voolstra

**Affiliations:** Department of Biology, University of Konstanz, Konstanz 78457, Germany; Department of Biology, University of Konstanz, Konstanz 78457, Germany; Department of Biology, American University, Washington, DC, 20016, USA; Computational Biology Research Center (CBRC) and Red Sea Research Center (RSRC), Biological and Environmental Sciences and Engineering Division (BESE), King Abdullah University of Science and Technology (KAUST), Thuwal 23955, Saudi Arabia; Department of Computer and Information Science, University of Konstanz, Konstanz 78457, Germany; Faculty of Information Technology, Monash University, 3168, Australia; Department of Biology, University of Konstanz, Konstanz 78457, Germany

## Abstract

**Summary:**

We present Coracle, an artificial intelligence (AI) framework that can identify associations between bacterial communities and continuous variables. Coracle uses an ensemble approach of prominent feature selection methods and machine learning (ML) models to identify features, i.e. bacteria, associated with a continuous variable, e.g. host thermal tolerance. The results are aggregated into a score that incorporates the performances of the different ML models and the respective feature importance, while also considering the robustness of feature selection. Additionally, regression coefficients provide first insights into the direction of the association. We show the utility of Coracle by analyzing associations between bacterial composition data (i.e. 16S rRNA Amplicon Sequence Variants, ASVs) and coral thermal tolerance (i.e. standardized short-term heat stress-derived diagnostics). This analysis identified high-scoring bacterial taxa that were previously found associated with coral thermal tolerance. Coracle scales with feature number and performs well with hundreds to thousands of features, corresponding to the typical size of current datasets. Coracle performs best if run at a higher taxonomic level first (e.g. order or family) to identify groups of interest that can subsequently be run at the ASV level.

**Availability and implementation:**

Coracle can be accessed via a dedicated web server that allows free and simple access: http://www.micportal.org/coracle/index. The underlying code is open-source and available via GitHub https://github.com/SebastianStaab/coracle.git.

## 1 Introduction

The role of prokaryotes becomes ever more important in the consideration of ecosystem- and organismal-level processes (McFall-Ngai *et al.* 2013; Peixoto *et al.* 2022). Although the assessment of bacteria that are present in a given environment or organism is now commonplace by means of 16S rRNA marker gene sequencing, the identification of bacterial communities and taxa that are indicative of or associated with a specific condition or setting is of particular interest. This provides a framework for biomarker development to aid environmental monitoring or identification of microbial candidates for prospecting in microbiome restoration/rehabilitation to increase host resilience or organismal well-being (Peixoto and Voolstra 2023). For instance, coral bleaching triggered by rising seawater temperatures is decimating coral cover globally (Hughes *et al.* 2017; Eakin *et al.* 2022). This spurs the development and application of active interventions to counter the ongoing decline (Voolstra *et al.* 2021a; Voolstra *et al.* 2023). Microbiome stewardship, the management of ecosystem function using the microbiome, e.g. through the provision of beneficial microorganisms that increase stress tolerance and improve recovery, is shown to work in principle (Rosado *et al.* 2019; Santoro *et al.* 2021), but the underlying mechanisms and the selection of putative beneficial microbes remain elusive (Peixoto *et al.* 2021, 2022). Further, the process of culturing and screening putative beneficial bacteria to be tested as probiotics can be laborious and time-consuming (Schultz *et al.* 2022; Dörr *et al.* 2023). To this end, artificial intelligence (AI)-based approaches have the potential to accelerate and optimize this process. This is because AI-based approaches allow pattern recognition in high-dimensional and big datasets (many features, many samples) that typically exceed the capabilities of simple statistics. For instance, machine learning (ML) can be applied to predict host phenotypes by human gut microbiome composition (Marcos-Zambrano *et al.* 2021) and guide precision medicine (Schork 2019). AI-based quantitative approaches to discovering potentially beneficial bacteria could therefore support the identification of putative coral probiotics by analyzing existing datasets. While research on biomarker identification and feature selection for multi-omics datasets exists, identification of bacterial communities associated with specific continuous variables of interest poses unique challenges. This is (i) because many existing methods are built for binary classification tasks that do not account for continuous data, but more importantly, (ii) because commonly conducted correlational analyses are limited to detecting linear relationships. ML approaches by comparison are superior for revealing nonlinear relationships and making accurate predictions from complex data. Physiological traits, such as thermal tolerance, are not “either-or” but rather are distributed as a continuum in natural populations (Evensen *et al.* 2022; Humanes *et al.* 2022). Thus, a framework for selection of bacteria associated with a “higher” or “lower” measurement of a continuous physiological variable is desirable. We have built Coracle, an ML framework that can work with complex features (e.g. microbial assemblage) and continuous target variables (e.g. thermal tolerance, growth, activity, resilience, etc.). Coracle is programmed to allow a robust identification of features associated with a given target variable even within smaller datasets. We show the utility of Coracle by running it on a 16S rRNA gene amplicon-based microbiome dataset with accompanying standardized thermal tolerance thresholds (i.e. ED50 values) from the same coral samples (Voolstra *et al.* 2020; Evensen *et al.* 2021, 2022, 2023). Potential other analyses can include identification of microbes associated with growth, disease susceptibility, recovery rate from injury, etc. Running Coracle in a successive manner at a higher (family) and then lower (Amplicon Sequence Variant, ASV or Operational Taxonomic Unit, OTU) taxonomic level identified bacterial taxa previously implicated in coral thermal resilience that can then serve as a starting point for further elucidation.

## 2 Implementation

**Figure 1.**
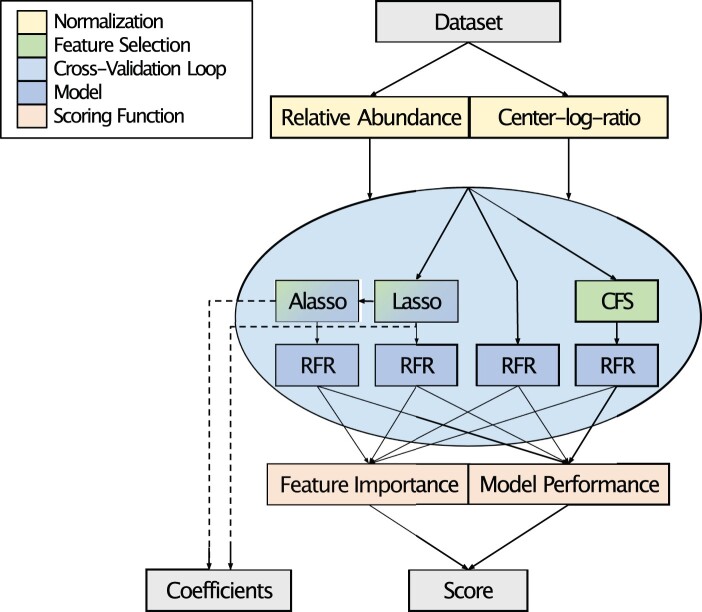
Coracle workflow. The Coracle workflow requires two input files (Dataset): one file that is a community data matrix, i.e. either ASVs with associated counts or aggregated counts at a higher taxonomic level, and a second file containing a continuous variable (e.g. physiological data such as thermal tolerance, growth, etc.) for the same set of samples. Data are then processed to assign each feature a score based on its importance with regard to the target variable of interest, weighted by the predictive performance of each model. The framework outputs the ranked list of all features along with their scores, sub-measures, and the coefficient values obtained from regression-like models. These coefficients indicate the direction of association (positive coefficients = an increase/decrease in bacterial abundance associated with an increase/decrease in the continuous variable; negative coefficients = an increase/decrease in bacterial abundance associated with a decrease/increase in the continuous variable). Abbreviations: RFR = Random Forest Regression, CFS = Correlation-based Feature Selection, LASSO = Least Absolute Shrinkage and Selection Operator, ALASSO = Adaptive LASSO.

This AI framework can select bacterial taxa associated with an increase or decrease in any continuous target variable (Fig. 1). To achieve this, the framework uses a multilevel ensemble approach that consists of multiple steps: In a first step, it forks through two popular forms of transformation (i.e. l1- and centered log ratio-normalization) (Aitchison 1982). After transforming the data, it applies various feature selection methods to extract a subset of features to feed into the ML models. Metrics of interest will then be aggregated via a scoring function to allow a simple yet informative ranking of bacterial taxa associated with thermal tolerance (i.e. ED50 values).

The task of identifying bacterial taxa or communities associated with a trait of interest (e.g. thermal tolerance) has several underlying complexities. Continuous target variables require regression-like models. In addition, bacterial community data are often sparse and high-dimensional, requiring methods to counter overfitting and the “curse of dimensionality” (i.e. arising difficulties when working with high-dimensional data, e.g. the amount of data needed to obtain statistical significance increasing exponentially with added feature counts). Therefore, the present framework validates the performance of all traversals via leave-one-out cross-validation, maximizing the training data for each cycle. With the typically small sample sizes (in comparison with the number of features) in multi-omics datasets, the leave-one-out cross-validation allows us to use the maximal number of samples for training (n-1) and consequently one sample for testing, without introducing overfitting. Coracle cycles through the dataset such that each sample will be used for testing once. While this comes at the cost of computational complexity, it can be considered more efficient/economical than the creation of bigger sample sets. As we only have two phases, training and validation, the parameters in the model cannot be optimized separately. Instead, the framework is optimized for an aggregated (collapsed) form of the microbiome. Based on training datasets, the taxonomic levels of “order” and “family” performed well and showed a similar feature number and resolution. Thus, parameters were broadly optimized for these two levels, although any other taxonomic level can be used as input to the framework, but may result in potential losses of robustness, performance, and an increased computational complexity. If the lowest taxonomic level (i.e. ASV or OTU level) is the desired output, prefiltering the data first is recommended to reduce runtime complexity. One promising alternative route for this is a two-tier approach with an identification of promising bacterial families (or orders) first, followed by a subsequent second Coracle run on the lowest level (ASV/OTU), but solely for the ASVs/OTUs of the previously identified high-scoring bacterial families (or orders).

Coracle follows an ensemble approach to support the robustness of feature selection, minimization of overfitting, and maximization of insights gained from available datasets. Two ensemble options can be considered: an ensemble of different models and an ensemble of different data subsets employing the same model. In Coracle, both approaches are implemented. For the implementation of an ensemble approach for the models employed, we chose a set of largely different approaches, including steps of preprocessing, feature selection, and ML to exploit their differing strengths and offset their weaknesses (Fig. 1). The first two transformation methods, l1-normalization and center log ratio-normalization (Aitchison 1982), are applied in parallel on bacterial abundance counts (community data matrix). Both are popular normalization methods for microbial abundance data and allow the examination of mathematically differing associations. Within the cross-validation loop, three feature selection methods, Lasso (Tibshirani 1996), adaptive Lasso (Zou 2006), and correlation-based feature selection (CFS) (Hall 2000), are applied. For the implementation of an ensemble approach using variable input data (Fig. 1), different subsets are utilized in the random forest regression step (Louppe 2014) to get an ensemble of regression trees. These are used to model subsets of selected features as well as the original feature set. Additionally, Lasso and adaptive Lasso are used to collect a number of feature coefficients to gain first implications on direction of association of selected bacteria taxa.

To aggregate all different steps and enable comparison of the different results, a scoring function combines the metrics of feature importance and model performance and ranks the bacterial taxa according to their score.

$$\mathrm{scor}e_{k}=\frac{\sum_{t=1}^{8}\;\;\mathrm{importanc}e_{kt}\;.\;R_{t}{}^{2}}{8}\;.\;\frac{m_{k}}{8}$$

**Figure 2.**
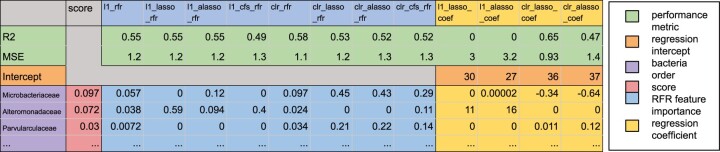
Coracle output. The first two rows show the *R^2^* and mean squared error metrics (green) for each Coracle model (column headers). The intercept for the regression models (orange) is presented in the third row. The intercept is useful to contextualize regression models, but not important to consider for interpretation of results. The following row headers are the input features (here: bacterial families; purple) ranked by their aggregated score (red). This score is the main outcome of Coracle and identifies the most important and robust features (here: bacterial taxa associated with thermal tolerance). The feature importance of the RFRs (blue) and the regression coefficients (yellow) allow to retrace the scoring and further analyze the output. This output is generated based on a previously published dataset that examined 28 coral colonies across 4 sites for their thermal tolerance and microbial assemblage (Voolstra *et al.* 2021b). Bacterial family assemblage at the baseline temperature (30°C) and colony-based ED50 thermal tolerance estimates were used as input files for Coracle to find bacterial taxa associated with thermal tolerance (Supplementary Data).

The respective importance of input feature *k* (i.e. bacterial taxa at the family/order or ASV/OTU level, depending on the taxonomic level chosen in the input file) in model *t* is represented by the *importance_kt_*, whereby $R_{t}^{2}$ represents the popular *R^2^* metric for model *t* and *{m_k_ ∈ ℕ | m_k_ ∈ [0,8]}* stands for the number of models that chose feature *k*. The final output includes all model performances, the feature importances, and coefficients of each feature (i.e. bacterial taxa at the family/order or ASV/OTU level, depending on the taxonomic level chosen in the input file), as well as the overall aggregated score, by which the resulting output is sorted (Fig. 2).

Given that typical biological datasets feature small sample sizes (<500 samples), computational complexity in this regard is of minor importance. However, the computational burden of running Coracle scales significantly with the number of features. Thus, the 2-tier approach consisting of an initial run at the family/order level (with aggregated ASV/OTU counts), followed by a subsequent run at the ASV/OTU level of those families/orders that produced high-scoring results, is advised for datasets featuring >1000 ASVs/OTUs.

It is important to note that while the performance evaluation requires leave-one-out cross-validation to prevent training and validating on the same data, the coefficients and feature importance are obtained via training the respective models on the whole dataset.

## 3 Application/case study

Our specific goal of this AI framework was to select coral bacterial taxa associated with tolerance/resilience to thermal stress (see also Fig. 1) (Supplementary File S1, S2). For a pilot run, we analyzed the association between bacterial assemblage (16S rRNA gene amplicon data) and thermal tolerance (ED50 standardized thermal tolerance values) of 28 *Stylophora pistillata* coral colonies across four Red Sea sites (Voolstra *et al.* 2021b) on the aggregated taxonomic level of bacterial family (Supplementary File S3). A partial view on the family-level output is given in Fig. 2. High scores for the bacterial family output were obtained for Microbacteriaceae (negative association), Alteromonadaceae (positive association), Vibrionaceae (positive association), Rhodobacteraceae (positive association), Flavobacteriaceae (negative association), and Endozoicomonadaceae (positive association) (among others) (Supplementary File S4), many of which were previously found associated with coral thermal tolerance (Rosado *et al.* 2019; Santoro *et al.* 2021; Savary *et al.* 2021; Voolstra *et al.* 2021b; Buitrago-López *et al.* 2023). ASVs underlying high-scoring families (only the top5 high-scoring families were considered) were then consecutively run in Coracle (Supplementary File S5) and produced high-scoring bacterial taxa (Supplementary File S6) that were previously deemed functionally important in coral microbiome studies, e.g. taxa of *Alteromonas* (Ziegler *et al.* 2017; Doering *et al.* 2021), *Marinobacter* (Camp *et al.* 2020), and *Aurantimicrobium* in the phylum Actinobacteria (Epstein, Torda and van Oppen 2019). Performance assessments based on the predictive capabilities of single models alone (i.e. paths in the Coracle workflow, Fig. 1) demonstrate the potential of the framework. Importantly, the assessments also showed that there is no single best path that outperformed all other approaches. With regard to the direction of association, the output of regression coefficients obtained from the Lasso models gives a first hint on the direction of association between the respective bacterial taxa and the physiological variable. The robustness of the Coracle output, i.e. whether the same taxa retrieve high-scoring outputs in iterative runs, was tested by running Coracle 100 times using the same input dataset. Considering the top10, top20, top30, top40, top50, and all taxa at the family and at the ASV level (ASVs of top5 families considered), the correlations between the feature’s score and rank across repeat runs were very high (>0.98 Pearson’s correlation coefficient for all instances; >0.93 Spearman rank correlation coefficient for all top scoring taxa; and >0.8 when considering all features) (Supplementary File S8). Sensitivity to sample size, which is a known limitation of random forests, was assessed via a jackknifing approach. To do this, we generated 10 randomly jackknifed datasets for each number of samples (*n* = 27, 26, 25, …, 5) to run Coracle (Supplementary File S7). Subsequently, we compared the average performance (mean *R*^2^) and standard deviation between each of the 10 datasets for any given number of samples. Coracle performance was good down to ∼20 samples and provided acceptable and still meaningful performance down to ∼10 samples, which one might consider a minimum sample size. In general, larger sample sizes will always be beneficial for performance and confidence. Based on these results, we recommend sample sizes ≥ 20 and encourage the use of Coracle at lower sample sizes with conscientious interpretation of the results.

## 4. Conclusion

Coracle is an AI framework that uses an ensemble approach of prominent feature selection methods and ML models to identify associations between bacterial communities and continuous variables. We tested and optimized Coracle using coral microbiome datasets. A typical use-case scenario is that a researcher is studying a continuous phenotypic characteristic (e.g. growth, stress tolerance, etc.) or variable of interest (e.g. salinity, turbidity, nutrient levels, etc.) while characterizing the microbiome to assess whether changes in bacterial community assemblage align with differences in the target variable (host physiology or environment). Coracle can identify bacterial taxa that are predictive of phenotypic trait or environmental condition performance, and thus provide a means to align host biology or the prevailing environment with microbiome assemblage. Notably, the application of Coracle is not restricted to microbial community data matrices but can process other types of high-dimensional data, such as gene expression matrices, in association with a continuous variable. Thus, Coracle can be used for a wide range of datasets with similar properties (a data matrix and a continous response variable for the same set of samples as input files). Importantly, Coracle can only account for association and not for causation. Thus, the obtained results need to be validated in real-world experiments. We set up a convenient web server implementation of the Coracle framework accessible at http://www.micportal.org/coracle/index. The web interface provides a tutorial for the upload of data files and execution of Coracle, including example files that can be downloaded for correct formatting of input data tables. The results are sent to the email address submitted on the webpage. The code of Coracle is accessible at https://github.com/SebastianStaab/coracle.git.

## Supplementary Material

btad749_Supplementary_Data

## Data Availability

The authors confirm that the data supporting the findings of this study are available within the article, its supplementary materials, at http://www.micportal.org/coracle/index, and at https://github.com/SebastianStaab/coracle.git.

## References

[btad749-B1] Aitchison J. The statistical analysis of compositional data. J R Stat Soc Series B Stat Methodol 1982;44:139–60.

[btad749-B2] Buitrago-López C , Cárdenas A, Hume BCC et al Disparate population and holobiont structure of pocilloporid corals across the Red Sea gradient demonstrate species-specific evolutionary trajectories. Mol Ecol 2023;32:2151–73. 10.1111/mec.16871. 36869609

[btad749-B3] Camp EF , Kahlke T, Nitschke MR et al Revealing changes in the microbiome of Symbiodiniaceae under thermal stress. Environ Microbiol 2020;22:1294–309. 10.1111/1462-2920.14935. 31997503

[btad749-B4] Doering T , Wall M, Putchim L et al Towards enhancing coral heat tolerance: a “microbiome transplantation” treatment using inoculations of homogenized coral tissues. Microbiome 2021;9:102. 10.1186/s40168-021-01053-6. 33957989 PMC8103578

[btad749-B5] Dörr M , Denger J, Maier CS et al Short-term heat stress assays resolve effects of host strain, repeat stress, and bacterial inoculation on Aiptasia thermal tolerance phenotypes. Coral Reefs, 2023, 42(6), 1271–1281. 10.1007/s00338-023-02427-y.

[btad749-B6] Eakin CM , Devotta D, Heron S et al The 2014-17 global coral bleaching event: the most severe and widespread coral reef destruction. Res Sq 2022. 10.21203/rs.3.rs-1555992/v1.

[btad749-B7] Epstein HE , Torda G, van Oppen MJH. Relative stability of the *Pocillopora acuta* microbiome throughout a thermal stress event. Coral Reefs 2019;38:373–86. 10.1007/s00338-019-01783-y.

[btad749-B8] Evensen NR , Fine M, Perna G et al Remarkably high and consistent tolerance of a Red Sea coral to acute and chronic thermal stress exposures. Limnol Oceanogr 2021;66:1718–29. 10.1002/lno.11715.

[btad749-B9] Evensen NR , Parker KE, Oliver TA et al The coral bleaching automated stress system (CBASS): a low‐cost, portable system for standardized empirical assessments of coral thermal limits. Limnol Oceanogr-Meth 2023;21:421–34. 10.1002/lom3.10555.

[btad749-B10] Evensen NR , Voolstra CR, Fine M et al Empirically derived thermal thresholds of four coral species along the Red Sea using a portable and standardized experimental approach. Coral Reefs 2022;41:239–52. 10.1007/s00338-022-02233-y.

[btad749-B11] Hall MA. Correlation-based feature selection of discrete and numeric class machine learning. (Working paper 00/08). 2000. Hamilton, New Zealand: University of Waikato, Department of Computer Science.

[btad749-B12] Hughes TP , Kerry JT, Álvarez-Noriega M et al Global warming and recurrent mass bleaching of corals. Nature 2017;543:373–7. 28300113 10.1038/nature21707

[btad749-B13] Humanes A , Lachs L, Beauchamp EA et al Within-population variability in coral heat tolerance indicates climate adaptation potential. Proc Biol Sci 2022;289:20220872. 36043280 10.1098/rspb.2022.0872 PMC9428547

[btad749-B14] Louppe G. Understanding random forests: from theory to practice. arXiv [statML], 10.48550/ARXIV.1407.7502, 2014, preprint: not peer reviewed.

[btad749-B15] Marcos-Zambrano LJ , Karaduzovic-Hadziabdic K, Loncar Turukalo T et al Applications of machine learning in human microbiome studies: a review on feature selection, biomarker identification, disease prediction and treatment. Front Microbiol 2021;12:634511. 33737920 10.3389/fmicb.2021.634511 PMC7962872

[btad749-B16] McFall-Ngai M , Hadfield MG, Bosch TCG et al Animals in a bacterial world, a new imperative for the life sciences. Proc Natl Acad Sci U S A 2013;110:3229–36. 23391737 10.1073/pnas.1218525110 PMC3587249

[btad749-B17] Peixoto RS , Sweet M, Villela HDM et al Coral probiotics: premise, promise, prospects. Annu Rev Anim Biosci 2021;9:265–88. 10.1146/annurev-animal-090120-115444. 33321044

[btad749-B18] Peixoto RS , Voolstra CR. The baseline is already shifted: marine microbiome restoration and rehabilitation as essential tools to mitigate ecosystem decline. Front Mar Sci 2023;10:1218531. 10.3389/fmars.2023.1218531.

[btad749-B19] Peixoto RS , Voolstra CR, Sweet M et al Harnessing the microbiome to prevent global biodiversity loss. Nat Microbiol 2022;7:1726–35. 10.1038/s41564-022-01173-1. 35864220

[btad749-B20] Rosado PM , Leite DCA, Duarte GAS et al Marine probiotics: increasing coral resistance to bleaching through microbiome manipulation. ISME J 2019;13:921–36. 30518818 10.1038/s41396-018-0323-6 PMC6461899

[btad749-B21] Santoro EP , Borges RM, Espinoza JL et al Coral microbiome manipulation elicits metabolic and genetic restructuring to mitigate heat stress and evade mortality. Sci Adv 2021;7:eabg3088 10.1126/sciadv.abg3088. PMC8363143 34389536

[btad749-B22] Savary R , Barshis DJ, Voolstra CR et al Fast and pervasive transcriptomic resilience and acclimation of extremely heat-tolerant coral holobionts from the Northern Red Sea. Proc Natl Acad Sci USA 2021;118(19):e2023298118. 10.1073/pnas.2023298118. PMC8126839 33941698

[btad749-B23] Schork NJ. Artificial intelligence and personalized medicine. Cancer Treat Res 2019;178:265–83. 31209850 10.1007/978-3-030-16391-4_11 PMC7580505

[btad749-B24] Schultz J , Modolon F, Rosado AS et al Methods and strategies to uncover coral-associated microbial dark matter. mSystems 2022;7:e0036722. 10.1128/msystems.00367-22. 35862824 PMC9426423

[btad749-B25] Tibshirani R. Regression shrinkage and selection via the Lasso. J R Stat Soc 1996;58:267–88.

[btad749-B26] Voolstra CR , Buitrago-López C, Perna G et al Standardized short-term acute heat stress assays resolve historical differences in coral thermotolerance across microhabitat reef sites. Glob Chang Biol 2020;26:4328–43. 10.1111/gcb.15148. 32567206

[btad749-B27] Voolstra CR , Peixoto RS, Ferrier-Pagès C. Mitigating the ecological collapse of coral reef ecosystems. EMBO Rep 2023;24:e56826. 10.15252/embr.202356826. 36862379 PMC10074092

[btad749-B28] Voolstra CR , Suggett DJ, Peixoto RS et al Extending the natural adaptive capacity of coral holobionts. Nat Rev Earth Environ2021a;2:747–62. 10.1038/s43017-021-00214-3.

[btad749-B29] Voolstra CR , Valenzuela JJ, Turkarslan S et al Contrasting heat stress response patterns of coral holobionts across the Red Sea suggest distinct mechanisms of thermal tolerance. Mol Ecol2021b;30:4466–80. 10.1111/mec.16064. 34342082

[btad749-B30] Ziegler M , Seneca FO, Yum LK et al Bacterial community dynamics are linked to patterns of coral heat tolerance. Nat Commun 2017;8:14213. 10.1038/ncomms14213. 28186132 PMC5309854

[btad749-B31] Zou H. The Adaptive Lasso and its Oracle Properties. J Am Stat Assoc 2006;101:1418–29.

